# Real-time decoding of covert attention in higher-order visual areas

**DOI:** 10.1016/j.neuroimage.2017.12.019

**Published:** 2018-04-01

**Authors:** Jinendra Ekanayake, Chloe Hutton, Gerard Ridgway, Frank Scharnowski, Nikolaus Weiskopf, Geraint Rees

**Affiliations:** a Wellcome Trust Centre for Interventional and Surgical Sciences, University College London, London, United Kingdom; b Wellcome Trust Centre for Neuroimaging, University College London, London, United Kingdom; c Institute of Cognitive Neuroscience, University College London, London, United Kingdom; d Siemens Molecular Imaging, Oxford, United Kingdom; e University of Oxford, Headington, United Kingdom; f Psychiatric University Hospital, University of Zürich, Lenggstrasse 31, 8032 Zürich, Switzerland; g Neuroscience Center Zürich, University of Zürich and Swiss Federal Institute of Technology, Winterthurerstr. 190, 8057 Zürich, Switzerland; h Zürich Center for Integrative Human Physiology (ZIHP), University of Zürich, Winterthurerstr. 190, 8057 Zürich, Switzerland; i Department of Neurophysics, Max Planck Institute for Human Cognitive and Brain Sciences, Leipzig, Germany

## Abstract

Brain-computer-interfaces (BCI) provide a means of using human brain activations to control devices for communication. Until now this has only been demonstrated in primary motor and sensory brain regions, using surgical implants or non-invasive neuroimaging techniques. Here, we provide proof-of-principle for the use of higher-order brain regions involved in complex cognitive processes such as attention. Using realtime fMRI, we implemented an online ‘winner-takes-all approach’ with quadrant-specific parameter estimates, to achieve single-block classification of brain activations. These were linked to the covert allocation of attention to real-world images presented at 4-quadrant locations. Accuracies in three target regions were significantly above chance, with individual decoding accuracies reaching upto 70%. By utilising higher order mental processes, ‘cognitive BCIs’ access varied and therefore more versatile information, potentially providing a platform for communication in patients who are unable to speak or move due to brain injury.

## Highlights

• Proof-of-principle of a ‘cognitive brain-computer-interface’ using realtime fMRI.
• Higher order visual brain regions used to decode the allocation of attention.
• Online single-block classification of 4-quadrant spatial attention to realworld images.
• Brain signal detection made more efficient by using ‘m-sequences’.
• Higher order mental processes produce more information for communication interfaces.

## Introduction

Brain-computer interfaces (BCIs) attempt to link measures of brain-related physiological activity with control of a device for communication or movement. A standard approach is to target brain activations produced in primary sensory or motor cortex (Jackson and Zimmermann, 2012), mapping the function of the target brain region with BCI output in a one-to-one fashion e.g. using motor cortical activations to control a hand prosthesis, or using retinotopic representations in primary visual cortex to direct a cursor on a screen (Andersson et al., 2013a, Birbaumer et al., 2008, Golub et al., 2016, Lebedev and Nicolelis, 2006, Miranda et al., 2015, Murphy et al., 2015). Cognitive BCIs seek to advance this premise by engaging higher-order brain regions, which control or combine basic afferent sensory information to produce behaviourally meaningful actions, or target regions which are involved in overarching processes such as attention (Richard et al., 2011, Tankus et al., 2014, Vansteensel et al., 2010, Wullimann et al., 2004). Visual attention is closely linked to visual awareness, acting to identify the location and semantic value of visual information. For cognitive BCIs linking higher-order mental processes with environmental interaction, attention provides an accessible cognitive process (Astrand et al., 2016, Astrand et al., 2014, Daliri, 2014, Tremblay et al., 2015). We used realtime fMRI (rt-fMRI) to test whether brain activations in higher-order visual cortex could be accurately classified in real-time (see also Data-in-Brief articles 1 & 2). Specifically, we examined brain activations that occur in relation to the control of covert shifts of spatial attention to stimuli representing real-world objects. In addition to utilising information linked to the control of attentional-shifts to spatial location and object category, we also added information related to the timing of the presentation of stimuli, by using m-sequences in each of the quadrants. We purposefully combined these different sources of information to enrich the BOLD signal produced by covert shifts of attention. By explicitly doing this, we sought to optimise classification accuracy, in line with our objective of providing proof-of-principle for a cognitive BCI.

Rt-fMRI enables concurrent analysis and online visualisation of fMRI data, a process normally performed offline (Cox et al., 1995). Once a particular cognitive process has been linked with a defined brain activation, neural activations can be converted into bits of information which serve as information transfer units for the BCI (Tehovnik et al., 2013). From here, there is no requirement for an explicit behavioural output, as the imaging data acts as a communication surrogate. An early example of this approach used brain activations produced by motor imagery, mental calculation and inner speech, to control letter selection on a virtual keyboard (Sorger et al., 2012). A more intuitive and attractive approach might be to identify brain activation produced by cognitive command signals, which specify a particular plan or action (Esterman et al., 2009).

We were specifically interested in identifying top-down signals, produced in higher-order visual cortex in relation to the control of attention. Top-down control is classically linked with spatial attention. It is enacted upon visual cortex by enhancing populations of neurones associated with retinotopically-represented regions of space in the outside world (Carrasco, 2011, Noudoost et al., 2010). Control of the allocation of visuospatial attention may additionally incorporate the biological importance of the stimulus being attended to (Vossel et al., 2014), with neural responses in brain regions lower down the visual hierarchy being modulated by contextual influences (Gilbert and Li, 2013, Gilbert and Sigman, 2007a). We examined 3 brain regions; parietal lobe, lateral occipital cortex (LOC), and fusiform face area (FFA), all of which have been suggested to contain salience maps (Gottlieb, 2007, Zenon et al., 2010), and have roles in integrating position and category-specific information (Carlson et al., 2011). LOC and FFA have been traditionally recognised as being object-selective cortex. They have also been shown to demonstrate retinotopy (Cichy et al., 2011a, Halgren et al., 1999, Kim and Biederman, 2011, Kim and Kastner, 2013, Saygin and Sereno, 2008a), as well as modulation by attention (Reddy et al., 2007, Yi et al., 2006). Parietal cortex has been suggested to have a more explicit role in top-down control, including mediating shifts of attention, control of salience maps, and object discrimination (Bressler et al., 2008, Chiu et al., 2012, Esterman et al., 2009, Gmeindl et al., 2016, Koenigs et al., 2009, Yantis et al., 2002). These regions may therefore act as sites of top-down modulation, or serve as ‘binding’ points for multiple sources of information, including object and spatial information. As a result, the neural activity produced in these regions may offer a high signal-to-noise ratio (Gattass et al., 2005, Sclar et al., 1990, Serences and Yantis, 2007) for the successful implementation of a BCI decoding command signals modulating higher order visual information linked to the allocation of visual attention (Andersson et al., 2011, Andersson et al., 2009, Astrand et al., 2014, Bahramisharif et al., 2010).

We hypothesised that signals linked to the covert allocation of spatial attention could be amplified by the inclusion of information related to the stimulus being attended to (i.e. object and feature-based information), and the timing of its presentation. To further increase BCI efficiency, we introduced quadrant-specific alterations of the temporal presentation of the stimuli. M-sequences, or *maximum shift L-level register sequences,* are pseudorandom sequences of integers which can be used to optimise stimulus presentation (Buračas and Boynton, 2002). They ensure that signals related to stimulus events presented close together in time can be optimally separated. We implemented this in order to further separate brain activations produced by attention to stimulus streams in a specific quadrant. Brain activations were separately extracted from bilateral FFA, LOC and parietal cortex. Quadrant-based parameter estimates were used in a winner-takes-all approach, to evaluate on a single-block basis, which location was being attended to. This work provides proof-of-principle for a real-time fMRI ‘attention-based’ BCI using higher order brain regions.

## Methods

### Participants

Eight healthy adult volunteers (24–32 years of age; mean age = 28 years, 4 females) with normal or corrected-to-normal visual acuity were recruited to participate in the experiment. Each participant provided written informed consent and the study was approved by the local ethics committee.

### Stimuli

The visual stimuli consisted of four categories: *faces, houses, body parts,* and *food/drink*. Faces and house stimuli were obtained from an in-house repository. Stimuli for body parts and food items were created using stock images. There were 16 unique exemplars per category per quadrant. Each stimulus subtended 2 degrees of visual angle in diameter, and was presented at an eccentricity of 6° from the centre of the screen. All images were rendered to ensure identical greyscale values, and mean luminance using a custom designed MATLAB (Mathworks, Natick, USA) script.

### fMRI scanning

Experiments were performed on a 3 T Allegra head-only scanner, using a standard transmit–receive head coil. Functional data were acquired with a single-shot gradient echo planar imaging sequence (matrix size, 64**_**64; field of view, 192_192 mm; isotropic resolution, 3 × 3 × 3 mm; 32 slices with ascending acquisition; slice thickness, 2 mm; slice gap, 1 mm; echo time (TE), 30 ms; repetition time (TR), 1920 ms; flip angle, 90°; receiver bandwidth, 3551 Hz/pixel). In the middle of each scanning session, double-echo fast, low-angle shot sequence (FLASH) field maps (TE1, 10 ms; TE2, 12.46 ms; resolution, 3 × 3 × 2 mm; slice gap, 1 mm) were acquired and used to correct geometric distortions in the images attributable to field inhomogeneities.

### Real-time set up

We used Turbo Brain Voyager (TBV, Brain Innovations, Maastricht, the Netherlands) with custom real-time image export tools programmed in ICE VA25 (Weiskopf et al., 2004a, Weiskopf et al., 2004b), and custom scripts running on MATLAB. The real-time data preprocessing was performed in Turbo Brain Voyager and encompassed 3D motion correction with realignment to a preselected template, smoothing (6 mm FWHM Gaussian kernel), incremental linear detrending of time series (128s high pass filter) and statistical parametric mapping. Participants’ brain activations blood oxygen level-dependent (BOLD) as region-of-interest (ROI) time course(s) were extracted from prescribed ROI masks. These were averaged and exported by TBV with a delay of 2s from the acquisition of the image. Images were corrected for the effects of head motion in realtime. Signal drift, spikes and high frequency noise were further removed in real time from the exported time courses with the custom MATLAB scripts (Koush et al., 2012).

### Optimising the timing of stimulus presentations using M-sequences

The timing of presentations for the stimuli in each quadrant was prepared using a quadrant specific m-sequence (Buračas and Boynton, 2002). Within a block each stimulus presentation represented an event, with each one lasting for 500 ms. The stimulus presentations for each quadrant were interspersed with a set number of blank stimuli in keeping with a quadrant-specific m-sequence (Fig. 2). The m-sequences were prepared to ensure maximum orthogonality, providing 32 stimulus presentation slots per quadrant per block, and optimising placement of ‘blank’ stimuli. Attention to each quadrant-specific stimulus stream would therefore produce quadrant-specific neural activity with distinguishable haemodynamic responses (Buračas and Boynton, 2002).

**Fig. 1:**
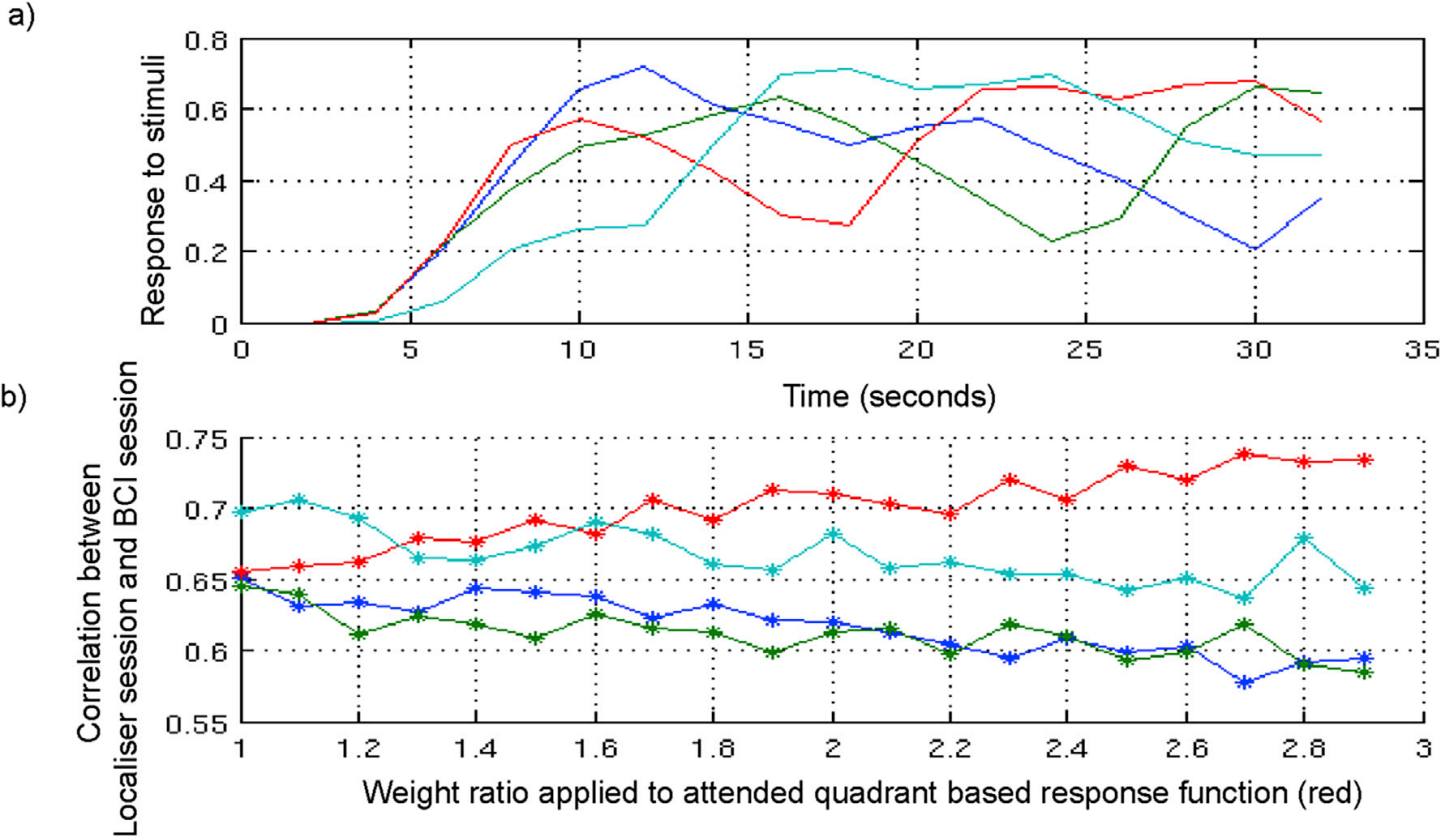
Graphs showing the modeled brain responses to m-sequences by convolving the HRF with the delta functions for the m-sequence for each quadrant. (a) Timeseries for each quadrant, showing the relative orthogonality for each quadrant. (b) Degree of correlation between the timeseries from the ‘localiser’ session with the ‘attended’ quadrant (red line) versus the other three simultaneous presented quadrant-based stimulus streams. By introducing a weighting to each of the quadrant time series, we examined if it would make it more discrete from the other three. The introduction of weighting served to mimic the effect of attention.

Prior to running the experiment, a simulation was used to confirm that expected BOLD signals for each quadrant could be distinguished as being different from the other three. The simulations were based on convolving m-sequence based stimuli with noise and the haemodynamic response function (HRF; Fig. 1). This was performed by generating four m-sequences that were uncorrelated, and convolving them with a canonical HRF. The frequencies were sampled down to the typical TR (i.e. approximately 2s, 15 data points for a 32s sequence). The response function produced simulated the BOLD signals during the localiser run i.e. when stimuli were being presented in one quadrant per block. This was repeated for each quadrant, and then put together to confirm that the simulated ‘timeseries’ were uncorrelated (Fig. 1).

**Fig. 2:**
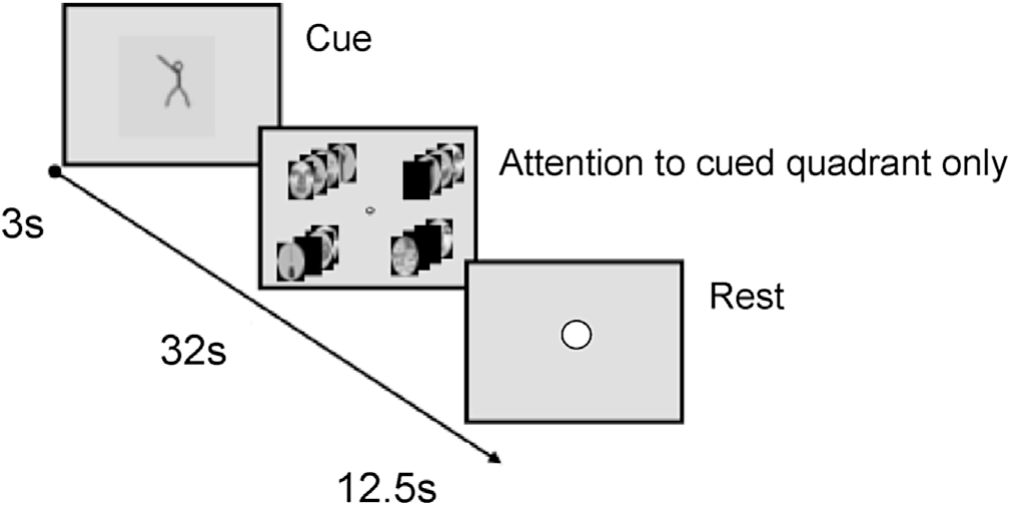
‘Cued attention’ session schematic. Participants were cued to attend stimuli presented in one quadrant per block. The directional cue stimulus was a stick man pointing towards the quadrant to be attended (first screen). During stimulus presentation in the 4 quadrants (i.e. second screen), blank stimuli (shown as black images) were interspersed with stimuli from the other four categories (i.e. faces, houses, body parts, food/drink), enabling quadrant-specific m-sequences to be used for stimulus presentation. During the rest block (i.e. third screen) participants maintained central eye fixation, facilitated by a white dot at the centre of the screen.

The random noise was initially generated from pseudorandom values drawn from a standard normal distribution, with a mean level of zero and standard deviation of 1. The standard deviation of the noise was then scaled by the ratio of the contrast to the noise level of the simulated response function to the contrast to the noise level of the simulated noise (assuming the same standard deviation for both response and noise). Finally the scaled noise was added to the response function.

The correlation coefficient between the individual simulated timeseries from the ‘localiser’ session and the combined simulated ‘timeseries for the ‘BCI’ sessions were calculated. The weighting of the contribution of one sequence (i.e. the 'attended sequence') to the total response was increased in small steps. These weights were normalised and acted to model the effect of attention on one of the quadrant-related timeseries in a BCI session. The correlation coefficient between the individual quadrant-specific timeseries from the localiser session and the same quadrant in the presence of the combined timeseries from the BCI session were calculated. The higher the correlation for a specific quadrant between the localiser session and the BCI session, the more separable the neural activity linked to the allocation of attention to that quadrant in the presence of competing stimulus streams. We performed this sequence one hundred times for each weighting level with the addition of random noise. The average response frequency was then calculated. Fig. 1b illustrates the modeled BOLD responses for each quadrant and the effect of ‘attention’ (i.e. increased weighting on a specific quadrant). This confirmed that the modeled BOLD responses for each quadrant could be distinguished as being different from the other three, motivating the choice of each of the 4 quadrant specific m-sequences.

### Experimental procedure

**Fig. 3:**
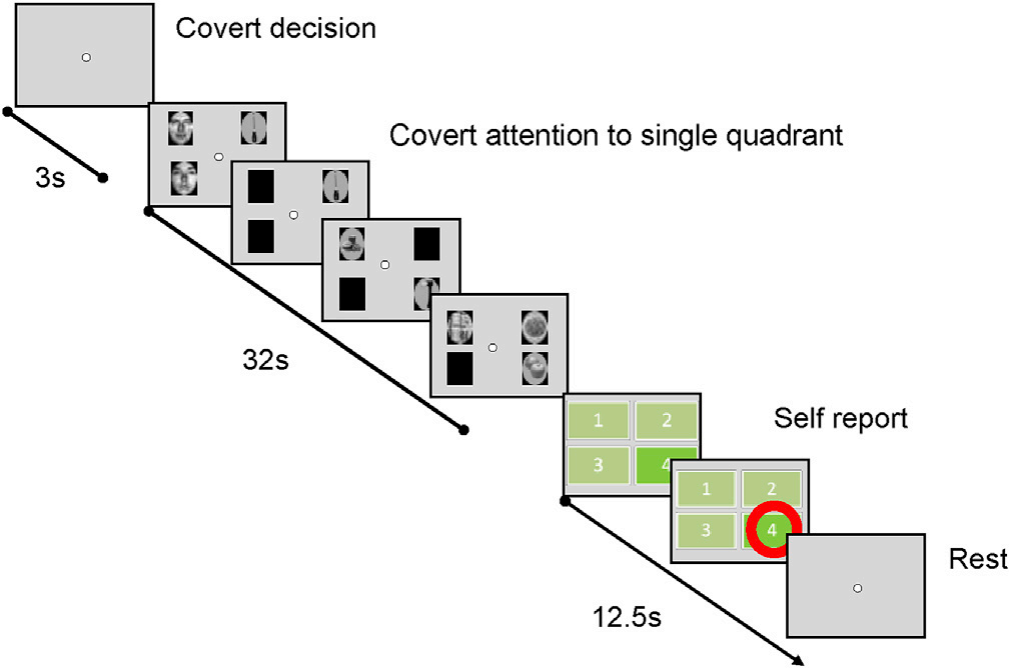
‘Non-cued’ sessions schematic. Participants were instructed to fixate centrally, and attend to one of four quadrants stimulus presentations for the duration of the block. They disclosed which quadrant they had attended at the end of each block using a button-box. Stimuli included four categories (faces, houses, household objects, body parts). ‘Blank’ stimuli (represented by black icons) appeared in a quadrant-specific fashion in keeping with a quadrant-specific m-sequence.

There were 5 sessions per participant. The structure of each session was the same. There were 8 blocks in each session, lasting 6 min 24s. The duration of one block was 48s, made up of 3s cue presentation, 32s of stimulus presentations, and 13s of rest. During the 32s of stimulus presentations, 32 stimuli were shown, together with 32 interspersed ‘blank’ intervals (400 ms per image, 100 ms inter-stimulus interval, 500 ms stimulus onset asynchrony). During this block of stimulus presentations, two ‘mini-blocks’ were shown each composed of 16 exemplars belonging to one of the 4 object categories (Fig. 2, Fig. 3). The order of the category of the mini-blocks was counter-balanced between and across sessions, and category exemplars were presented in a pseudo-random manner.

Participants were explicitly instructed to attend one quadrant per block. During the cued session, the sequence of cues was different each time for the first four blocks; this sequence was then repeated for the remaining 4 blocks. During the un-cued sessions (3–5), they were instructed to attend a different quadrant each time for the first four blocks, and to repeat this sequence in the subsequent four blocks.

### Session 1- localiser

During the first session, stimulus streams were presented in one quadrant of the screen for the duration of one block, with each quadrant hosting stimulus blocks twice per session. Participants were instructed to maintain central eye fixation throughout the session, and attend to the quadrant showing stimuli.

### Defining functional regions of interest

ROIs were selected using the TBV ROI selection option. For each participant, regressors for each stimulus category were placed at the onset of a stimulus block, for the duration of the bock, and were then convolved with the canonical HRF. The resulting parameter estimates were used to calculate a t-statistic at each voxel, indicating evidence of task-related activation. We used a t-threshold of 3. To define bilateral fusiform face area (FFA) voxels we contrasted parameter estimates evoked by faces against rest (t-contrast: faces > rest), and delineated the ROI in relation to ventral and lateral surfaces of the temporal lobe in proximity to the fusiform gyrus. To define bilateral lateral occipital cortex (LOC) voxels we contrasted parameter estimates evoked by objects against rest (t-contrast: everyday objects > rest), and delineated the ROI along the posterolateral aspect of the fusiform gyrus, extending ventrally and dorsally. For bilateral parietal regions we contrasted parameter estimates evoked by all stimuli versus rest. Using the Juelich histological atlas to provide anatomical landmarks (Eickhoff et al., 2006, Eickhoff et al., 2005), we selected voxels in the superior parietal lobe (SPL) and those on the dorsal and ventral banks of intraparietal sulcus (IPS; both regions which have been shown to demonstrate object-sensitivity (Kim and Biederman, 2011, Serences et al., 2004, Wojciulik and Kanwisher, 1999)). The t-maps were overlaid onto cortical hemispheres using TBV. Participant-specific functional ROIs were delineated manually and resulted in discrete selection of non-overlapping voxels in bilateral parietal cortex, FFA and LOC. (Please also see Supplementary results for ROI centroids).

### Session 2 - cued attention

During stimulus presentation blocks, stimuli were presented repeatedly and simultaneously in all four visual quadrants (Fig. 2). Attention to a particular quadrant was indicated using a directional cue presented during the cue interval. Each quadrant was cued twice per session. Participants were instructed to maintain central eye fixation throughout the session. To ensure participants remained engaged in all sessions, a button press was required if two successive exemplars were identical (i.e. one-back task). This occurred between one to three times per mini-block. All quadrants, in addition to the attended one had repeated stimuli. The n-back task was included to help attentional engagement through the presentation of stimuli. All button presses were taken to be associated with the attended quadrant.

### Sessions 3–5–BCI ‘decoding’ sessions

Stimuli were presented as described in the previous paragraph. Participants were now instructed to covertly attend a quadrant of their choice for the duration of a whole block while maintaining central eye fixation. They were further instructed to use a strategy that would enable them to attend all quadrants twice over the course of the scanning session. They disclosed the attended quadrant using a button press during the rest period at the end of each of block (Fig. 3).

### Eye tracking

Eye-tracking during fMRI was not performed in this experiment, due to the complexity of the experimental set-up. Eye movements could represent a potential confound – eye movement-related brain activations in the cortical oculomotor network may overlap with those produced by covert shifts of spatial attention (Corbetta et al., 1998). However, eye movements typically disturb decoding of attention, reducing classification accuracy to below chance (Gunduz et al., 2012, Treder et al., 2011). We used eye tracking in a non-realtime fMRI version of this experiment and obtained similar classification accuracies in the same brain regions to those generated with online decoding, with an absence of excessive eye movements (see data in brief article 2). Eye position was found not to vary in a consistent manner during the experiment, precluding fixations on attended quadrants.

### Analysis of main experiment (sessions 2 to 5)

We investigated the extent to which functionally delineated higher-order visual cortex ROIs could be used to predict the direction of spatial attention. The inclusion of unique temporal information in the presentation of stimuli at each of the four quadrant spatial locations was applied to improve decoding accuracy. The resulting accuracies for individual ROI based classifications were based on comparing the highest quadrant specific parameter estimate with the disclosed covertly attended quadrant during a task block.

Cortical responses to the four attentional conditions were specified using HRF-convolved regressors at the onset times of the images, together within a given m-sequence. Each m-sequence was unique and specific to each of the four quadrants; the same m-sequence for a given quadrant was used across all sessions, irrespective of the object category. A general linear model (GLM) modeled each of the quadrant parameter estimates over each block consisting of 24 vol ‘Decoding’ was carried out at the end of each block in a ‘winner-takes-all’ approach, based on which one of the four parameter estimates had the greatest mean value. Data were read by the script and lagged behind image acquisition by approximately 2s.

The attended quadrant, during a specific block, was the one with the highest representative parameter estimate. A prediction was made on a block-by-block basis, which could then be compared to the actual quadrant attended to by the participant (as indicated by the button-response at the end of each block) allowing decoding accuracies to be calculated across all sessions and blocks for all ROIs.

### Reaction times

The potential effects of the time taken during BCI usage and its effect on decoding accuracy are an important consideration for ensuring accuracy in a BCI. A possible effect of time might be to decrease decoding accuracy as a result of increasing fatigue with task performance over time. We therefore further examined the changes in decoding accuracy over time. We did this by dividing each session into the first four blocks and the second four blocks, and compared reaction times during the performance of an n-back task between the first half and the second half of the session. The reaction time data from two participants was corrupted, and was therefore not analysed.

## Results

### Decoding accuracies

**Fig. 4:**
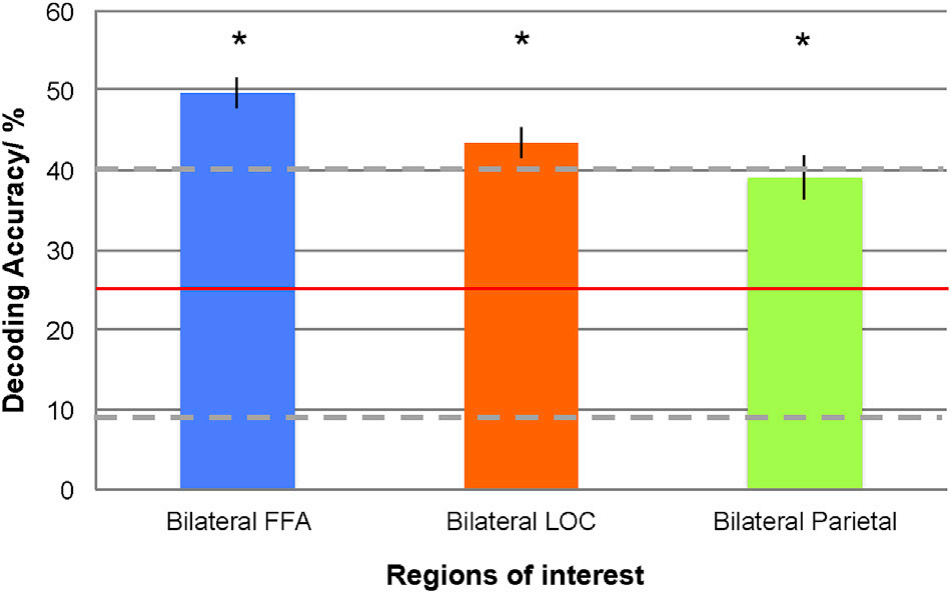
Participant-averaged decoding accuracy for the three ROIs averaged across sessions and blocks. Chance-level decoding at 25% (horizontal red line). Error bars indicate ±1 SEM. Dotted horizontal grey lines indicate confidence intervals. Asterisks indicate when decoding accuracy was significantly above chance.

We first examined decoding accuracies across all sessions and blocks, for each of the three bilateral ROIs (Fig. 4), to establish whether the quadrant to which attention was being directed could be decoded at above chance levels from signals evoked in each ROI. For each ROI (FFA, LOC, parietal), decoding accuracy was significantly above chance levels (25%): FFA (Decoding accuracy = 49.61, SD = 5.65, t (7) = 12.32, p < .001); LOC (Decoding accuracy = 43.36, SD = 5.40, t (7) = 9.63, p < .001); Parietal (Decoding accuracy = 39.06, SD = 7.83, t (7) = 5.08, p < .01).

**Fig. 5:**
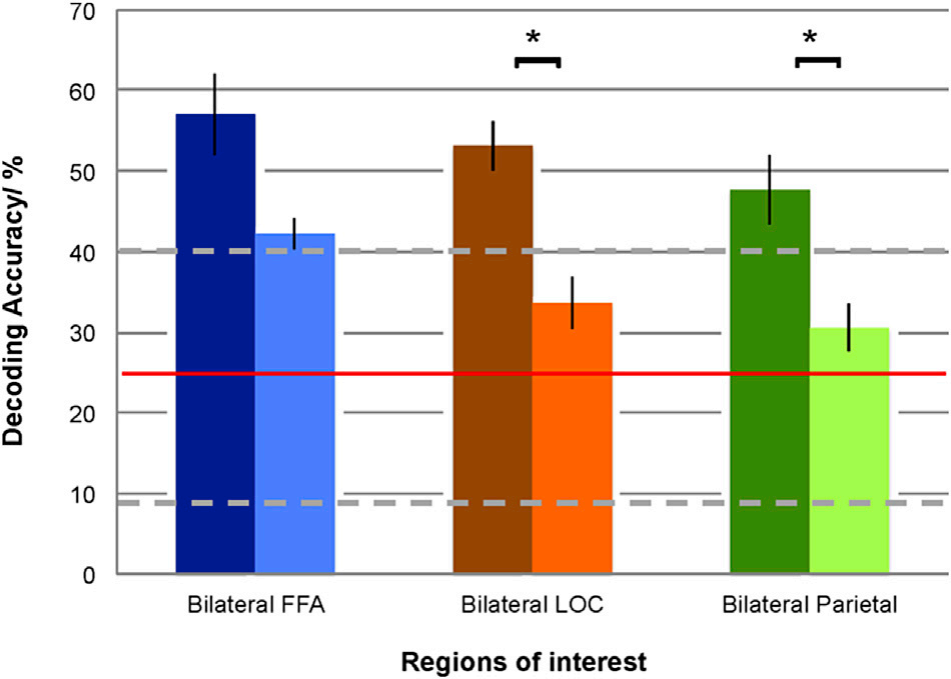
Decoding accuracies during each session, shown as pairs of bar graphs, comparing the first four blocks with the second four blocks. Chance is at 25% (horizontal red line). The columns in dark/solid colours represent decoding accuracy over the first four blocks, averaged across all sessions; the lighter columns represent decoding accuracy over the second four blocks, averaged across all sessions. Decoding accuracy in bilateral LOC and bilateral parietal ROIs was significantly higher during the first half of each session, as compared to the second half of each session. Error bars indicate ±1 SEM. Dotted horizontal grey lines indicate confidence intervals. Asterisks indicate significant differences in decoding accuracy, comparing the first four with the second four blocks.

**Fig. 6:**
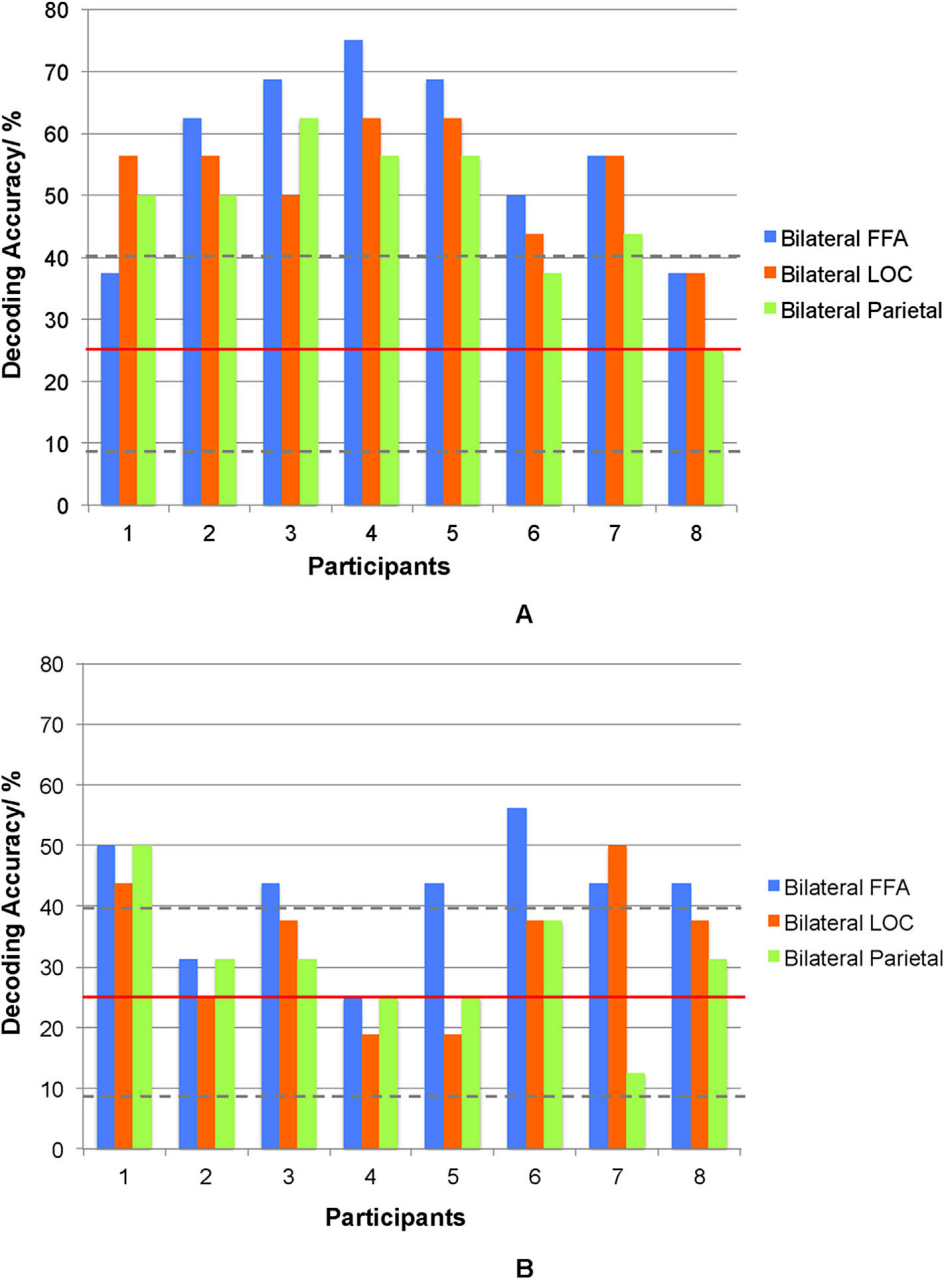
Decoding accuracies for individual participants, comparing the first four blocks (Figure. A), with the second four blocks (Figure. B), averaged across all sessions. Chance is at 25% (horizontal red line). Dotted horizontal grey lines indicate confidence intervals.

We had an a priori hypothesis that decoding accuracies would decrease with time as a result of fatigue. We hypothesised that this would be more likely to occur within sessions, rather than across sessions, which allowed for a rest between sessions (Fig. 5, Fig. 6). A paired *t*-test (2-tailed) comparing decoding accuracy over the first four blocks as compared to the second four blocks revealed a significant decline in decoding accuracy for bilateral LOC (t = 3.16, p = .016) and bilateral parietal ROIs (t = 2.94, p = .022). There was no significant decline in decoding accuracy for bilateral FFA (t = 1.92, p = .097) (Fig. 5).

### Data-driven assessment of statistical significance

We performed permutation testing to confirm the statistical significance of the classification accuracies for ROIs averaged across subjects. Predictions were repeatedly shuffled and compared with the correct allocation of attention in order to generate a data-driven distribution of classification accuracies under the null hypothesis. Permutation p-values were derived using percentiles. 10,000 permutations were carried per ROI per subject. Classification accuracies for all 3 ROIs were found to be statistically significant e.g. Bilateral FFA p = .0019 (individual participants p < .0025); Bilateral LOC p = .014 (individual participants p < .017); Bilateral Parietal p = .035 (individual participants p < .045).

### N-back task

Average accuracy for n-back task performance (e.g. number of accurate identifications of repeats in the attended quadrant) across the 6 participants on whom data was obtained was 64% (SD = 14%). The average false alarm rate was 17% (SD = 14%).

**Fig. 7:**
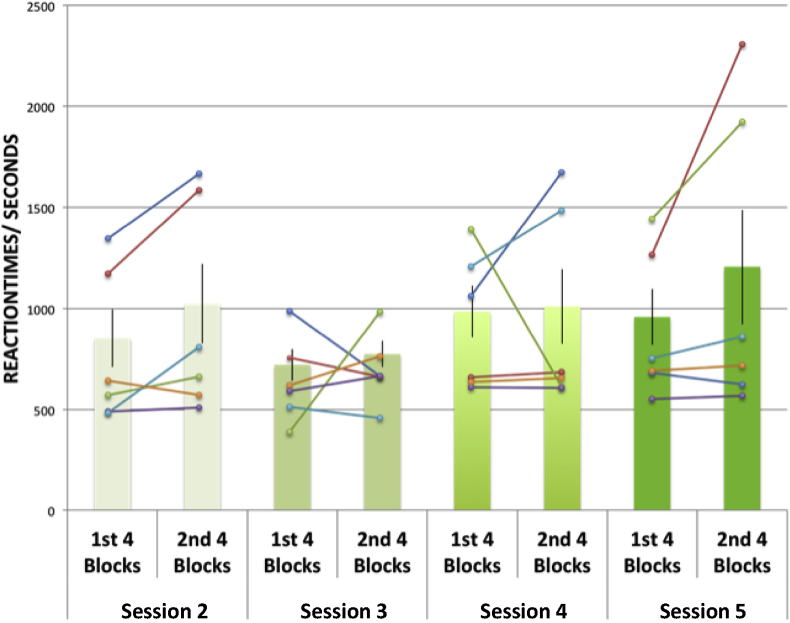
Graph showing average reaction times averaged across participants for n-back task performance, for each session. Sessions were divided further into the first 4 and second 4 blocks to show the effects of experimental time on task performance. Matched average reaction times for individual participants are shown for first 4 blocks and second 4 blocks of each session, using coloured connected lines for each participant.

An assessment of reaction times on the n-back task was also performed on the 6 participants on whom data was obtained, using an ANOVA across sessions (2–5) and blocks (averaged over first 4 blocks, averaged over second 4 blocks) (Fig. 7). A change in reaction times affecting task performance either across and/or within sessions (i.e. across blocks) would be suggestive of fatigue as a result of time. A main effect of block was observed (F (1,5) = 7.751, p = .04), with an increase in reaction times over the blocks (Fig. 7). There was no effect of sessions (F (3,15) = 1.00, p = .42), nor was there an interaction of blocks with sessions (F (3,15) = 0.49, p = .70).

### Offline eye-tracking

We used eye tracking in a non-realtime fMRI ‘offline’ version of this experiment. Similar classification accuracies were obtained in the same brain regions to those used in the current study, with a concurrent absence of excessive eye movements (see Data in brief article 2).

Participants were instructed to maintain central eye fixation throughout all the 8 task blocks, for each of four ‘decoding’ sessions. A repeated measures ANOVA was performed on the X and Y eye position data separately, and the factors of horizontal attention (left, right) and vertical attention (up, down) demonstrated no main effect of horizontal or vertical attention, and no interaction between them: for X-position data: left vs. right, F (1,7) = 0.697, p = .431; up vs. down, F (1,7) = 0.387, p = .554, interaction, F (1,7) = 1.164, p = .316; for Y–position data: left vs. right, F (1,7) = 0.697, p = .431, up vs. down, F (1,7) = 0.387, p = .554, interaction, F (1,7) = 1.164, p = .316. Participants therefore did not significantly move their eyes in a consistent manner over the experiment. Furthermore, we found overall decoding accuracies in the standard fMRI version of this experiment were comparable (Bilateral parietal 39% cf. 39%; Bilateral LOC 50% cf. 43%; Bilateral FFA 47% cf. 50%).

We further investigated whether there were systematic differences in eye position in relation to the attended quadrant. A two-way ANOVA with factors of quadrant (four levels, upper left, upper right, lower left, lower right) and sessions (four levels, 2–5) revealed no main effect of session or quadrant for the X and Y eye positions and X and Y standard deviations (see Table 1).

**Table 1:** Table showing results of statistical tests performed on eye position data taken during an offline experiment with the same procedural set-up as the reported online experiment examining realtime decoding of attention. Greenhouse-Geisser corrections were applied following violation of sphericity.

	Session	Quadrant	Session × Quadrant
X-mean position	P = 0.23 F (1.29, 9.09) = 1.69	P = 0.41 F (1.25, 8.76) = 0.86	P = 0.19 F (2.02,1.50) = 0.19
Y- mean position	P = 0.55 F (2.03, 14.22) = 0.63	P = 0.12 F (1.59,11.15) = 2.66	P = 0.30 F (1.33,1.46) = 1.33
X-standard deviation	P = 0.51 F (1.26, 8.89) = 0.58	P = 0.34 F (3.90,3.40) = 0.34	P = 0.41 F (2, 14.01) = 0.41
Y-standard deviation	P = 0.39 F (1.01, 7.08) = 0.83	P = 0.13 F (1.63,11.38) = 2.54	P = 0.32 F (1.14, 7.95) = 1.18

## Discussion

We report a novel rt-fMRI-based cognitive BCI based on the online classification or ‘decoding’ of the voluntary deployment of covert attention to spatially distinct streams of real-world stimuli. This study was inspired by the seminal work conducted with electroencephalography (EEG)-based BCIs using the P300 signal, a neurophysiological correlate of attention (Birbaumer et al., 2000, Donchin et al., 2000, Farwell and Donchin, 1988, Piccione et al., 2006). Here, we exploited the increased spatial resolution of fMRI. We sought to optimise quadrant-specific decoding for the purposes of an operational BCI, which might work by providing different user-options at each of the 4 quadrant locations. Classification or ‘decoding’ of the visual responses in the three target brain regions was therefore driven by combined contributions from top-down attentional modulation signals, as well as category-specific stimulus information, and the timing of stimulus presentation. M-sequences were used to optimally distinguish BOLD signals, by producing quadrant-specific timing for the stimulus streams (see also supplementary discussion, and Data in brief articles 1 & 2 for preceding work). A novel algorithm was implemented with a ‘winner-take all’ decision rule using quadrant-specific parameter estimates. Decoding accuracies in selected higher-order visual ROIs (i.e. FFA, LOC, parietal cortex) were significantly above chance in all 3 ROIs (*p's* < 0.001); individual decoding accuracies reached between 60% and 70% during the first half of each experimental session. Participant reaction times on an interposed n-back task increased in the second half of each session, suggesting fatigue may have contributed to the observed reduction in decoding accuracies towards the end of each experimental session.

Attention enables focused processing of sensory signals evoked by environmental stimuli. Specific populations of neurones respond to the volitional direction of attention to circumscribed regions of space (which are retinotopically represented), or to real-world objects. Objects may also spatiotopically activate category-specific cortex (Saygin and Sereno, 2008b). Although specific cortical circuits subserve different aspects of attentional control (Corbetta et al., 2000, Hopfinger et al., 2000, Kastner et al., 1999, Pinto et al., 2013), there is a significant degree of overlap (Cichy et al., 2011b, Larsson and Heeger, 2006). This may enable one or more higher-order regions to generate an ‘attentional command signal’, biasing spatial and non-spatial features, and integrating emotional and motivational valence via an attentional priority map (Bisley, 2011). The outside world is spatially represented by internally maintained retinotopic maps, demonstrated throughout the visual hierarchy, including the dorsal (IPS) (Saygin and Sereno, 2008b) and ventral processing streams (e.g. LOC; Cichy et al., 2011a, Cichy et al., 2011b). An attention map is likely to be based on retinotopic representations, with specific top-down weighting of salient locations (Baluch and Itti, 2011), and an interaction between top-down and bottom-up influences (Bisley, 2011, Corbetta and Shulman, 2002). The increase in the functional weighting of the attended location by higher order brain regions may itself be linked to suppression of salient but behaviourally distracting stimuli at non-attended locations (Ipata et al., 2006).

Topographical information linking object position with retinotopic maps can be identified in higher-order regions traditionally associated with feature and category-based attention, e.g. FFA, LOC (Schwarzlose et al., 2008). Therefore, object category and retinotopy may be jointly coded in higher-order visual cortex (Corbetta et al., 1998, Gunduz et al., 2012, Larsson and Heeger, 2006). Allocation of a top-down attention command signal in these regions could act to co-ordinate separate category and spatial properties of a stimulus, in preparation for a behaviourally relevant action.

We used stimuli that would be relevant for day-to-day communication in a BCI for assistive communication (see also Data in brief article 2). Users would potentially be able to ‘indicate’ their requests to carers via images on a visual display e.g. a particular body part that needed medical attention, to request a food item, or ask for an individual using a facial image. In our study each quadrant provided a specific stream of information, which the participant could direct their attention to as required. These stimuli activated category-specific neural representations in higher-order visual cortex, specifically LOC, FFA and parietal lobe, making an additional contribution to brain activations produced by attentional shifts. Of note, previous rt-fMRI based decoding of category-based attention only used whole brain classifiers (Niazi et al., 2014). We also added a temporal element to help further distinguish haemodynamic responses produced by deploying attention to quadrant-specific streams of stimuli. Blank stimuli were interspersed with stimulus presentations, enabling the application of m-sequences to specify optimal event ordering. M-sequences are nearly orthogonal to cyclically time-shifted versions of themselves (Buračas and Boynton, 2002), affording maximal statistical efficiency for separating different stimulus events.

Our study provides proof-of-principle for a *cognitive* BCI, delivering classification accuracies for four-quadrant spatial attention deployment at approximately twice chance (i.e.25%)- FFA (50% accuracy, SD 5.65), LOC (43% accuracy, SD 5.40), Parietal lobe (39% accuracy, SD 7.83). Most BCIs use binary classifications e.g. left versus right (Kelly et al., 2005). The choice of rt-fMRI for a non-invasive BCI was based on its superior spatial specificity as compared to other non-invasive imaging modalities e.g. magnetoencephalography (MEG)/EEG (Sitaram et al., 2007). Andersson et al. used primary retinotopic cortex for a rt-fMRI BCI, decoding spatial attention at 7 T. Participants covertly directed attention to a high contrast grating or a high luminescence arrow (Andersson et al., 2013b, Andersson et al., 2012, Andersson et al., 2011, Andersson et al., 2010, Andersson et al., 2009). Accuracy for four-quadrant decoding reached 79% on average. However this was with simple high contrast stimuli. An important distinction with our BCI set-up was the use of higher-order brain regions and real-world stimuli. Higher order cognitive processes may be harnessed for a more versatile BCI (Friedrich et al., 2014, Tankus et al., 2014, Vansteensel et al., 2010). This may be necessary for BCI use in certain clinical populations. These include patients with amyotrophic lateral sclerosis (ALS) (Marchetti et al., 2013), a progressive disease of lower and upper motor neurones which ultimately leads to complete paralysis, and brain injury patients (Chen et al., 2011), where damage may only involve primary somatosensory cortex. Cognitive function and central control is preserved in these patients.

**Fig. 8:**
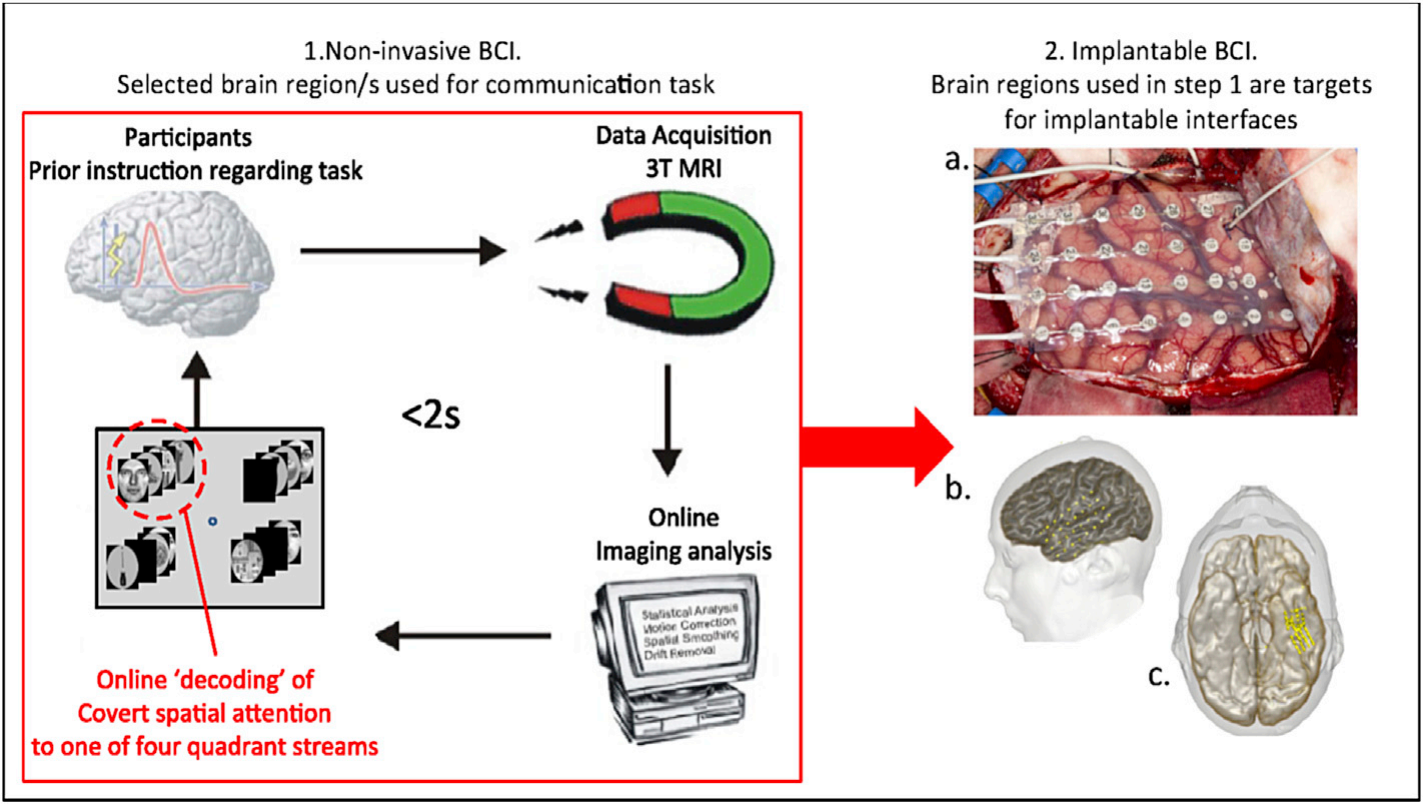
Proposed pipeline using a non-invasive BCI interface with rt- fMRI to prime and prepare specific brain regions with a BCI task, prior to surgery for placement of longer-term implantable BCI. 1) Realtime-fMRI decoding pathway (e.g. as used in this study) 2) A. Implantation of subdural electrodes (Image courtesy of Anna Miserocchi and Andrew McEvoy) B, C. 3D reconstruction showing final placement of temporal and inferior temporal subdural (ECoG) grids for recording of relevant cortical activity, as part of a long-term implanted BCI.

Previous BCI approaches utilising higher-order brain regions have focused on using brain activations that are *unrelated* to the task. Instead, they have been used as a surrogate for navigation e.g. through a virtual maze, or letter selection on an online keyboard (Sorger et al., 2012, Yoo et al., 2004). We targeted a cognitive process - spatial attention, which can be used to intuitively bypass explicit movement. Further, we specifically selected putative control regions with the aim of identifying concentrated neural populations in discrete cortical locations which may have multiple functional outputs (i.e. ‘multiplexing’; Gilbert and Sigman, 2007b, Ipata et al., 2006, Moxon and Foffani, 2015). In contrast to the use of large areas of brain to extract signal for a BCI, using smaller cortical areas engaged in cognitive control processes (Hauschild et al., 2012) may enable a higher signal-to-noise ratio by reducing the incidence of unrelated brain activations. A alternative approach would be to use pattern recognition techniques to improve information extraction i.e. whole brain classifiers (Niazi et al., 2014). However in the ultimate translation to a surgically implanted BCI (see Fig. 8), using a smaller region of brain facilitates use of a smaller prosthesis, minimising surgical exposure, reducing operative time, surgical risk and inpatient stay.

Implantable BCIs (e.g. extradural or intradural surface electrode strips, microelectrode arrays (MEAs)) offer advantages in terms of higher fidelity signal extraction and portability (Allison et al., 2007, Daly and Wolpaw, 2008, Wolpaw, 2012). More specifically, a ‘hybrid’ approach, combining use of a non-invasive BCI, such as rt-fMRI to allow tailoring of parameters prior to implantation with an intracranial BCI device, might provide an important means of optimisation of BCI performance. This is particularly important for the successful uptake and use of BCIs in clinical populations (e.g. ALS), where patients are more frail, and prone to fatigue during learning associated with BCI use (Riccio et al., 2013, van Gerven et al., 2009).

Recent work with implanted interfaces in primates has demonstrated sustained BCI use is associated with significant cortical reorganisation, resulting in the alteration of directional neuronal tuning properties of BCI-specific brain regions, and concurrent reduced modulation in BCI-adjacent neuronal populations (Ganguly et al., 2011, Orsborn and Carmena, 2013). Data extraction from a specific cortical location using a rt-fMRI based BCI could therefore be optimised by training with a non-invasive BCI such as that described in this study. This could then be followed by surgical implantation of a prosthesis in the target brain region with a higher likelihood of success. Fig. 8 illustrates a possible operational pipeline.

Recent proof-of-principle for this type of pipeline using primary sensory regions was demonstrated, using a rt-fMRI BCI to train retinotopic regions prior to intracranial recordings with electrocorticography (ECoG), for spatial attention deployment (Andersson et al., 2011). ECoG BCIs recording from primary visual cortex in non-human primates have demonstrated classification accuracy of >90% for attention to two spatial locations, and 67–79% for four locations (Astrand et al., 2014, Rotermund et al., 2013). MEAs have been used to classify a two-position spatial attention task using local field potentials from Macaque area MT (Esghaei and Daliri, 2014, Seif and Daliri, 2015). However MEAs cause brain tissue reactions, which limit the size of the implant that can be used, and affect signal stability and implant lifetime. Implantable BCIs can provide potential benefits as a result of closer proximity to the brain, although challenges remain with regards to long-term use and signal optimisation (Murphy et al., 2015, Obien et al., 2015).

In our study, classification accuracies for deployment of four-quadrant spatial attention were between 40 and 50% across subjects, but reached just above or just below 70% in individual participants. The latter is a level previously suggested as the operational accuracy required for use of BCIs in communication (Halder et al., 2013, Kübler et al., 2004, Kübler et al., 2001, Kübler et al., 1999). This level of accuracy (or near it) was only achieved during the first half of the experimental sessions, and only by some participants. There was a significant reduction in decoding accuracy during the second half of the experimental sessions. A majority of participants exhibited above chance classification in the first half of each scanning session, across the three ROIs (e.g. Participant 4, Fig. 6A), but performed less well during the second half of the experiment (Fig. 6B). Possible reasons for this decline in within-session decoding accuracies may have been related to fatigue (Assmus et al., 2003, Coull and Nobre, 1998). Reaction times were examined as a surrogate for fatigue, and were found to significantly increase within a session. Mental fatigue, linked to impairment in complex task performance, has been associated with reductions in BOLD activation (Assmus et al., 2005). Additionally, fatigued individuals are prone to distraction (Faber et al., 2012), as might have been caused by the use of multiple streams of stimuli. Therefore, ensuring sessions are short, e.g. 3–4 min may help to improve rt-BCI user-performance. Other potential experimental details, which may have affected decoding accuracy, relate to the visual stimuli themselves. We controlled for specific stimulus properties such as luminescence and grey scale values. On the other hand, local contrast differences between stimuli were not explicitly controlled for. Although this may have acted to reduce decoding accuracy, it was felt to more accurately represent the conditions and constraints of a real-world operational BCI set-up. Finally placing the quadrant-based stimuli more eccentrically may have helped to improve decoding accuracy.

Variations in decoding accuracy between individuals were observed among the three different ROIs used in the study, with some participants performing better with one ROI during the first half of the experiment, and another ROI during the second half of the experiment. The order of presentation of category of stimuli was balanced across quadrants, and over sessions to prevent biasing towards a particular category in a particular quadrant. The need to optimise ROI selection for classification in relation to communication-based BCIs using realtime fMRI has recently been addressed through the use of automated ROI selection on a per participant basis, combining a localiser, together with unsupervised machine learning algorithms (Lührs et al., 2017). Further participant-specific factors such as strategies used to allocate, control and maintain attention to particular quadrants are likely to vary, in addition to intrinsic differences in cognitive capacities and arousal (Ghose and Maunsell, 2002, Matthias et al., 2010, Willems et al., 2015). Other sources of variance may arise from unrelated fluctuations in the measured BOLD signal e.g. participant movement in the scanner.

A more sophisticated means of ensuring optimal BCI performance might be to actively feedback a measure of performance as an operant goal e.g. decoding accuracy (deBettencourt et al., 2015), or the level of brain activation in BCI-relevant regions (Andersson et al., 2012, Andersson et al., 2011). This type of closed-loop adaptive BCI may allow the user to monitor successful use of the BCI within a session, while facilitating instrumental neuroprosthetic learning, leading to improved BCI performance with successive use.

## Conclusions

This study demonstrates accurate decoding of attention-based information, using realtime fMRI. We accessed internal, higher-order processes which are not dependent on motor or primary sensory cortex activation and achieved decoding which reached 70% accuracy in some participants.

For a BCI to have perfect ecological validity it needs to satisfy two conditions–1/a user environment that reflects real world decisions and/or utilises common themes or stimuli 2/an operational mechanism which mimics or is similar to an actual neural process. We have attempted to address both of these requirements by i) using an overarching cognitive process (category-based and spatial attention) which can produce utilisable output in the context of a BCI and ii) accessing this process in a behaviourally meaningful way though the use of stimuli with real-word significance. (e.g., the selection of an item such as a glass of water from several objects that are presented in a spatially distributed manner). The application of m-sequences served to take advantage of underlying patterns in timing-related changes in cerebral blood flow. It is a statistical adjunct that enhanced our decoding approach, without acting as the principal driver. This study embodies the principles that are essential for the creation of an ecologically valid BCI, serving as the basis for further development.

A non-invasive BCI approach may provide a necessary first step to accessing important higher order brain regions in the pathway to implementing long-term implantable BCIs for applications such as aiding with communication in patients lacking the ability to move or speak.

## Conflicts of interest

All authors declare no financial or non-financial conflicts of interest.
