# Supplementary material for: Real-time decoding of covert attention in higher-order visual areas
Source: Neuroimage. 2018 Apr 1;169:462–72. doi: 10.1016/j.neuroimage.2017.12.019 (PMC5864512; doi:10.1016/j.neuroimage.2017.12.019)
Supplement: Supplementary Information [file mmc1.docx]

**Supplementary Table 1:**

**Table showing x,y and z co-ordinates for region-of-interest (ROI) centroids selected and used for online decoding.**

**(FFA: Fusiform Face Area, LOC: Lateral Occipital Cortex, Par: Parietal).**

| **ROI** | **PARTICIPANTS** | | | | | | | |
| --- | --- | --- | --- | --- | --- | --- | --- | --- |
|  | 1 | 2 | 3 | 4 | 5 | 6 | 7 | 8 |
| FFA | -36,-68,-20 | -34,-74,-14 | -34,-72,-12 | -40,-72,-16 | -36,-66,-18 | -32,-68,-6 | -52,-68,-8 |  |
| FFA | 34,-70,-20 | 36,-64,-18 | 26,-74,-12 | 36,-78,-16 | 38,-64,-12 | 38,-58,-6 | 40,-50,-24 | 36,-66,-18 |
| LOC | -44,-72,-2 | -40,-82,12 | -40,-82,10 | -50,-74,6 | -50,-78,2 | -46,-70,8 | -54,-64,0 | -44,-76,6 |
| LOC | 42,-72,0 | 42,-82,4 | 34,-82,10 | 48,-76,6 | 42,-74,2 | 32,-80,10 | 44,-70,0 | 38,-78,0 |
| Par | -30,-60,46 | -30,-56,46 | -34,-56,42 | 32,-58,58 | -34,-60,52 | -36,-60,52 | -36,-56,62 | -36,-72,24 |
| Par | 18,-56,48 | 26,-56,46 | 26,-54,42 | -34,-58,58 | 32,-62,56 | 30,-60,48 | 42,-48,48 | 30,-72,24 |

**Supplementary discussion**

We have previously conducted a 4-quadrant covert spatial attention study, using simple stimuli (left or right handed black and white spirals), without the application of m-sequences to designate stimulus presentations. Bilateral Occipital, Parietal and LOC ROIs were used. When applying univariate classification approaches no significant decoding was found for the deployment of 4- quadrant covert spatial attention (versus 25% chance): **Bilateral occipital** 24% p<0.78 t(12)=-0.81, **Bilateral LOC** 25% p<0.59 t(12)=-0.23, **Bilateral parietal** 23% p<0.94 t(12)=-1.64. Of interest, we applied multivariate classification approaches using simple linear classifiers (Linear Discriminant Analysis (LDA) classifier with and without principle components analysis (PCA), Correlational classifiers (CC)) – significant decoding was observed with all classifiers in all regions examined, although the values were comparatively low. e.g.: **Bilateral occipital**: 4 directions classification accuracy; **CC**: 30% p<0.01 t(12)=4.74, **LDA**: 31% p=0.01 t(12)=2,78, **LDA with PCA**: 30% p<0.01, t(12)= 3.12. **Bilateral LOC**: **CC**: 31% p<0.01 t (12)= 3.90, **LDA**: 30% p=0.04 t(12)=1.96, **LDA with PCA**: 28% p=0.02, t(12)= 2.39. **Bilateral Parietal**, **CC**: 29% p=0.02 t (12)=2.44, **LDA with PCA**: 27% p=0.08, t(12)= 1.53. This result indicated that decoding of spatial attention was possible, although the applicability of this to a BCI that would be able to decode to an operationally useful level would require optimisation. We have submitted this experiment and the associated results as a one of two contemporaneous Data in brief articles (referred to as Data in brief article 1).

As presented in the Data in brief article 2 we then examined the decoding of covert spatial attention to real-world stimuli using primary retinotopic cortex. Building on the previous work presented in the Data in brief article 1, M-sequences were used to specify stimulus presentations. Classification accuracies using a univariate approach of identifying parameter estimates modelling quadrants as specified by m-sequences yielded significant classification in non-category specific **Bilateral V2** and **Bilateral V3** of 28% (p<0.039, t(7)=2.53 versus 25% chance) and 30% (p<0.038, t(7)=2.55 versus 25% chance) respectively. By decoding from retinotopic ROIs, we looked at areas that would not be expected to be involved in category-specific attention-related processes. The resulting improvement of classification accuracies was sufficient to achieve statistical significance, but remained low. This suggested that m-sequences were supportive *but not sufficient* for a classifier decoding more ‘real world’ stimuli.

In this incremental approach, by next using higher order visual regions, which were also involved in the processing of category specific information linked to the stimuli being used, we observed an increase in decoding accuracies i.e. **Bilateral FFA** 47% p<0.004 t(7)=3.67, **Bilateral LOC** 50% p<0.001 t(7)=4.71, **Bilateral parietal** 39% p<0.023 t(7)=-2.41. This increase was indicative of a 0.75-1.5x increase in decoding accuracy, which might be attributed to the contribution of categorical information to decoding accuracy.
